# 30.1 IMMUNE PATHOGENESIS OF PSYCHOSIS: THE CHALLENGE OF CO-MORBIDITY

**DOI:** 10.1093/schbul/sby014.122

**Published:** 2018-04-01

**Authors:** Rachel Upthegrove, Carl Krynicki, Annalisa Gordianno, Carmine Pariante, Toby Rowland, Nicholas Barnes, Steven Marwaha, Benjamin Perry, Paola Dazzan, John Deakin

**Affiliations:** 1 University of Birmingham; 2 Institute of Psychiatry, Psychology and Neuroscience, King’s College London; 3 University of Warwick; 4 University of Manchester

## Abstract

**Background:**

The immune pathogenesis story of schizophrenia is gathering momentum, with increasing evidence from genetic, circulating biomarker and neuropathological studies. New treatment approaches are being trialled. However immune dysfunction is not unique to schizophrenia, and circulating proinflammatory biomarkers identified in schizophrenia have also been identified in bipolar disorder and major depressive disorders. Similarly, in recent times there has been an increasing recognition of commonality across categorical diagnoses at a symptom level; as RDoC criteria acknowledge. For example, depressive symptoms are common in schizophrenia, hallucinations and delusional beliefs common in mood disorders and anhedonia a cross diagnostic challenge

**Methods:**

This presentation will include data of altered circulating pro-inflammatory markers from recently completed meta-analysis in first episode psychosis, established schizophrenia and bipolar disorder, highlighting the potential pluripotent inflammation pathway to mental disorders and outline a circulating cytokine profile at the onset and development of mental disorder as related to symptom specific profiles.

**Results:**

Data on circulating inflammatory makers as related to symptom profiles cross-sectional and longitudinally will also be presented from the recently concluded NIHR funded BeneMin (The Benefits of Minocycline on negative symptoms in early phase psychosis) study.

**Discussion:**

Future research should recognise co-morbidity, adopt a dimensional approach, or investigate symptom specific biomarkers at early stages of illness with numbers large enough to explore an immune specific clinical profile. This knowledge is essential in the developing story of inflammation and psychosis with the most potential in decades to translate into tailored effective treatment options.

